# From virtual to real: comparison of field experiments and DEM simulation of twin-disc granular fertiliser broadcaster

**DOI:** 10.1038/s41598-026-39552-x

**Published:** 2026-02-12

**Authors:** Fırat Kömekçi, Vedat Demir, Ceren Kömekçi, Tuncay Günhan, Damla Doğu

**Affiliations:** Agricultural Machinery and Technologies Engineering, Faculty of Agriculture, Ege University, 35100 Bornova, Izmir, Turkey

**Keywords:** Agriculture machinery, Comparative analysis, Disc peripheral speed, Discrete element method, Distribution, Spreader, Engineering, Mathematics and computing

## Abstract

Twin-disc fertiliser broadcasters are commonly used to distribute granular fertiliser. The performance of these machines depends on various design and operational variables. Assessing the effects of these variables under real-world conditions requires extensive, time-consuming, labour-intensive, and material-intensive studies and analyses. The Discrete Element Method (DEM) approach enables computer-aided simulation of these processes. However, to ensure that simulation studies correspond with field studies, material properties must be used in the software with high accuracy, and simulation models must be selected appropriately for the operating conditions. The aim of this study was to compare field experiments and DEM simulations of fertiliser distribution with twin-disc fertiliser broadcaster. The field experiments were carried out with granular fertiliser and five different disc peripheral speeds according to the ASAE S314.4 and TS 2541 standards. For the DEM simulations, the twin-disc fertiliser broadcaster, the granular fertiliser, and the hoppers used in the field experiments were modelled, and the simulation experiments were conducted as a function of the operating parameters of the fertiliser broadcaster. In the field and simulation experiments carried out with three vanes, an average difference of 2% between the fertiliser quantities was determined at a 16 m effective working width.

## Introduction

Granular materials, such as agricultural products, solids, soil particles, gravel, coal, and rock, consist of grains in contact with surrounding air pores. The mechanical behaviour of granular materials is therefore inherently unstable and heterogeneous. The Discrete Element Method (DEM) has proven to be the most effective modelling technique for systems exhibiting discrete or particulate behaviour of granular materials in many scientific and engineering applications^1,2^.

The discrete element method (DEM) employs an explicit numerical scheme in which particle interactions are tracked through contact motion. By numerically solving Newton’s laws of motion, DEM enables the calculation of particle forces, trajectories, and velocities, which are updated iteratively until termination conditions are satisfied. From these dynamics, parameters such as collision frequency, dwell time, and impact energy can be derived, providing a basis for interpreting experimental results^1,3^.

Granular materials, generally characterised by rigid and inelastic particles with negligible thermodynamic effects, exhibit behaviour strongly influenced by their microstructure^4^. DEM allows realistic modelling of this behaviour by incorporating the random distribution of particle size and shape^5,6^.

Over the past three decades, DEM has been widely applied in agricultural engineering, particularly to centrifugal fertiliser broadcasters. Simulation models have been developed to describe particle motion on the disc and in flight, and to analyse the effects of fertiliser properties such as size, friction coefficient, and restitution coefficient on distribution patterns^7–13^. Complementary studies introduced control strategies, such as optical feedback-based feed gate adjustment, to improve uniformity of spreading^14–16^.

More recent work has systematically examined broadcasters with variable application rates, combining DEM simulations with experimental validation. These studies demonstrated that distribution patterns are influenced by design and operational parameters, such as vane inclination and disc height, which directly affect the uniformity and coefficient of variation of spreading performance^17–21^.

Zinkeviciene et al.^22^ and Bivainis et al.^23^ compared experimental and simulation results for the application of organic granular fertiliser with a disc fertiliser broadcaster. Bivainis et al.^23^ reported that it is possible to operate at high application rates and forward speeds by making small changes to the shape, length, and rotation angles of the vanes of a conventional fertiliser broadcaster. Zinkeviciene et al.^22^ found that the distance covered by the fertiliser granules depends in part on particle size, initial speed, fertiliser type, and the parameters of the rotating discs of the broadcaster. Distribution uniformity experiments with a disc fertiliser broadcaster are time-consuming, labour-intensive, and costly. It is possible to improve the uniformity of fertiliser distribution while saving time, labour, and costs by using DEM simulation.

This study examined the utility of DEM in field trials with a twin-disc fertiliser broadcaster by simulating its operation. This approach enables both machine manufacturers and testing authorities to gain significant insights into the machine by conducting simulations with appropriate data, rather than relying on labour-intensive trials.

## Materials and methods

**Fig. 1:**
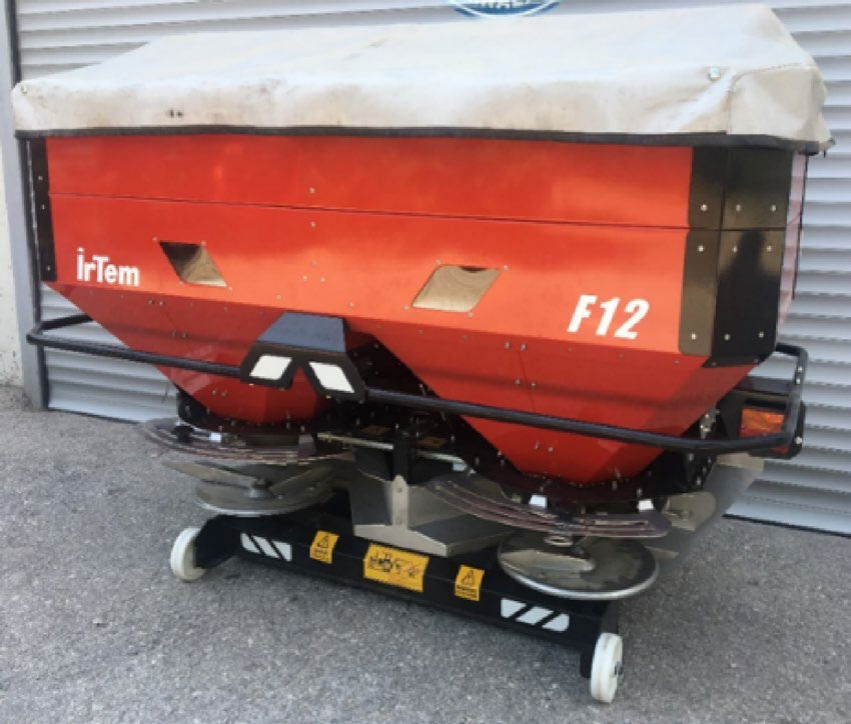
Twin-disc fertiliser broadcaster used in the study^24^.

In the study, a twin-disc fertiliser broadcaster was used (Fig. 1). The main fertiliser tank of the machine holds 1200 L, and the distribution discs are made of stainless steel with a diameter of 460 mm.

**Fig. 2:**
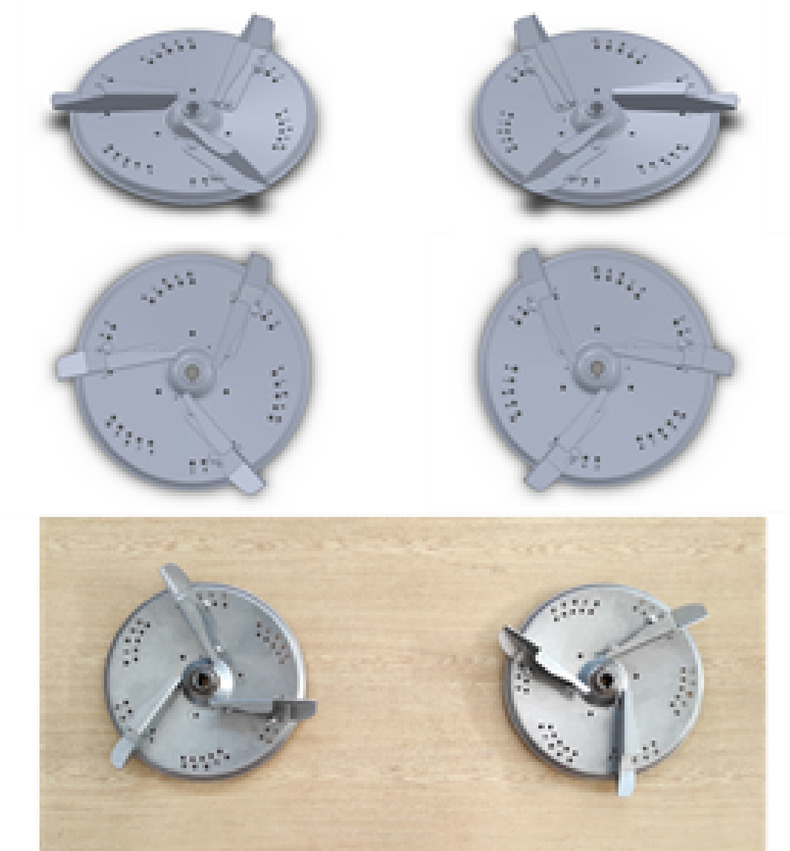
3D models and produced distributor discs with three vanes at an angle of 43°.

In the experiments, 230 mm long vanes were placed on the discs in sets of three. The discs were provided with five pinholes so that the vanes could be set at different working angles. In the study, the vanes were placed at an angle of 43° using pinhole number 3 (Fig. 2). Granular fertiliser with a solid density of 1600 kg m^−3^ was used in the experiments, and the results of the sieve analysis are shown in Table 1.

**Table 1:** The results of the sieve analysis for granular fertilizer^24^.

Sieves (mm)	Distribution (%)
4.00	12.57
3.35	82.98
2.00	4.12
1.00	0.33

### Field trials

The fertiliser application rate is set using an adjustment arm that can be moved along the guide channel beneath the hopper, which is graduated at equal intervals. Before starting fertiliser application, fertiliser was dropped onto the disc at a fixed opening (15 cm^2^), and the application rate was measured for each unit.

The uniformity of fertiliser distribution in the transverse direction by the twin disc fertiliser broadcaster was assessed outdoors in windless conditions, in accordance with ASAE S341.4^25^ and TS 2541^26^. The outdoor tests were conducted using a New Holland TM175 tractor. At the start of the tests, the fertiliser tank was filled to full capacity. During the tests, which were conducted at a fixed application rate, the fertiliser spreader discs were positioned 85 cm above and parallel to the ground, and the tests were performed at an average driving speed of 8 km h^−1^. During the experiments, the air speed and direction were measured at a height of 1.5 m above the ground before each test and none of the experiments were conducted at wind speeds above 2 m s^−1^^25,26^. In the tests on the uniformity of fertiliser distribution, the average air speed in the area passing through the centre of the arranged area was measured at 1.2 m s^−1^, and the ambient temperature was 40 °C. Air speed measurements were taken using a vane-type anemometer. The anemometer’s measurement range is 0–20 m s^−1^, with a measurement accuracy of ± 0.1 m s^−1^.

**Fig. 3:**
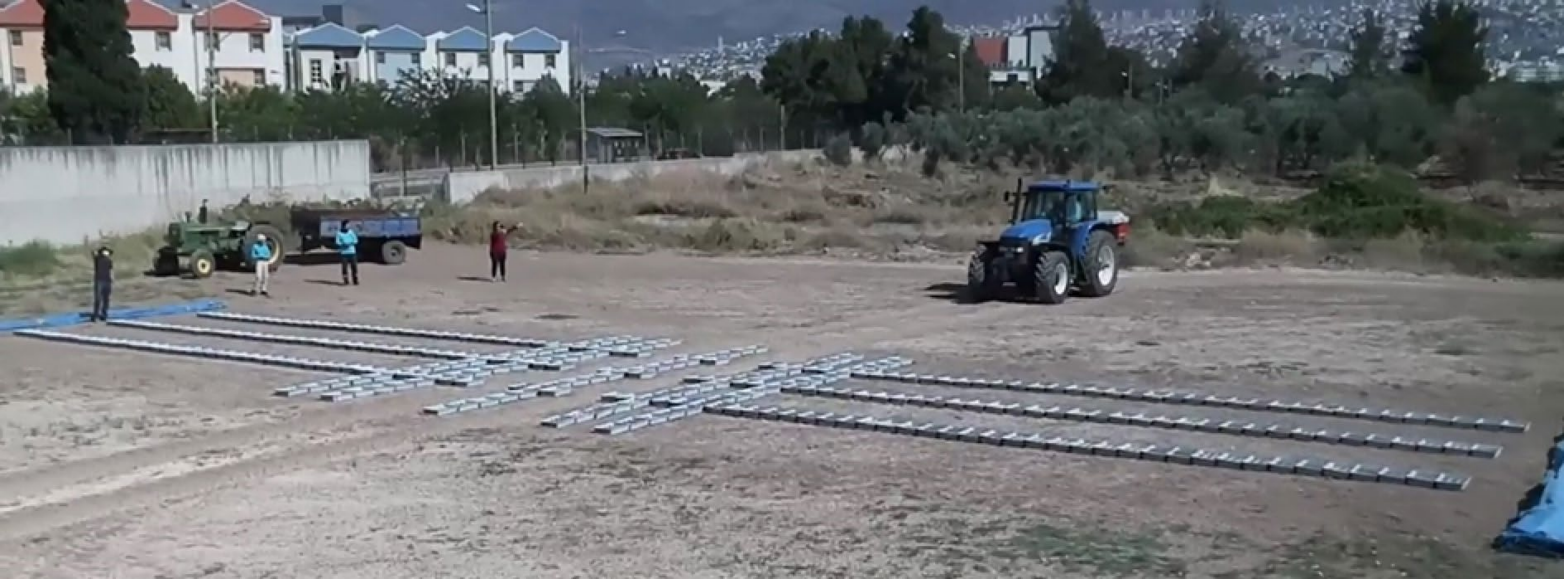
Arrangement of the collection containers in field trials^24^.

Before starting the experiments, the collection bins were arranged in three rows in a two-dimensional matrix on the field according to the ASAE S341.4 standard, ensuring three repetitions in the forward direction. A distance of 2000 mm was left between the rows. The fertiliser was collected in 495 × 330 × 110 mm bins, spaced equally on all three axes, with 42 bins on the right, 42 on the left, and 3 under the tractor. A distance of 800 mm was left between the collection bins in each row to allow the tractor wheels to pass between the bins during the tests (Fig. 3).

The field experiments were conducted with a cover opening that provided a fixed fertiliser rate of 300 kg ha^−1^, with three vanes at an angle of 43°, and five disc peripheral speeds of 13.85, 15.05, 16.26, 17.46, and 18.67 m s^−1^. After fertiliser application, the fertiliser collected in the bins was weighed on a precision scale.

### DEM analyses

**Fig. 4:**
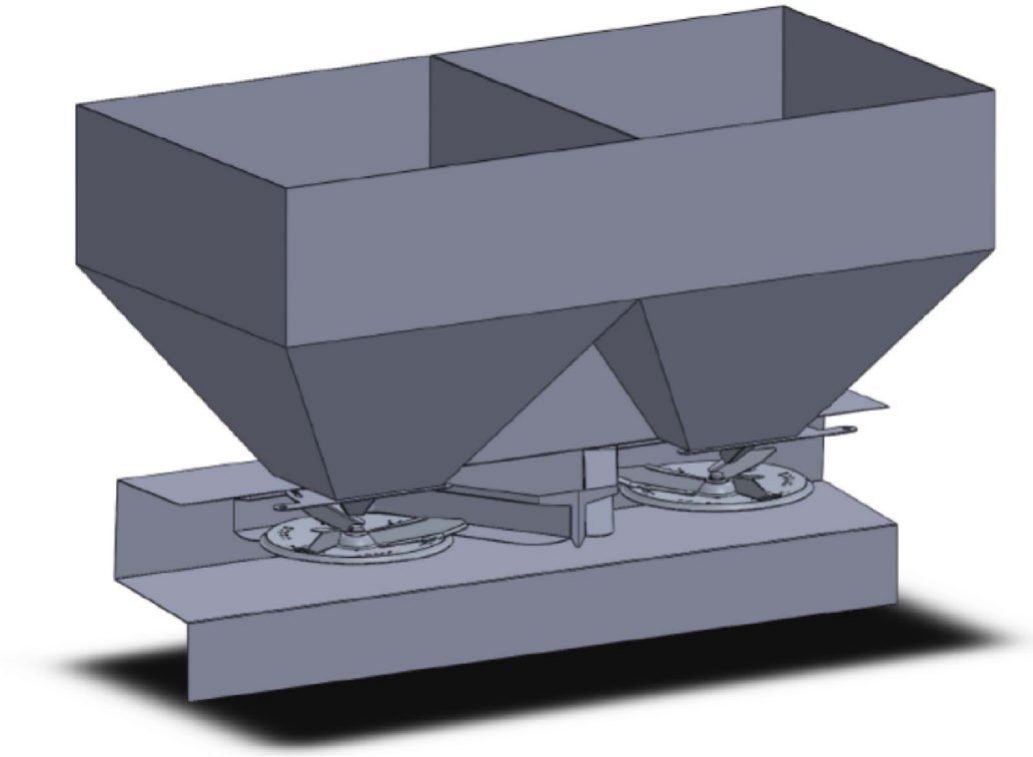
3D model of a twin-disc fertiliser broadcaster.

**Fig. 5:**
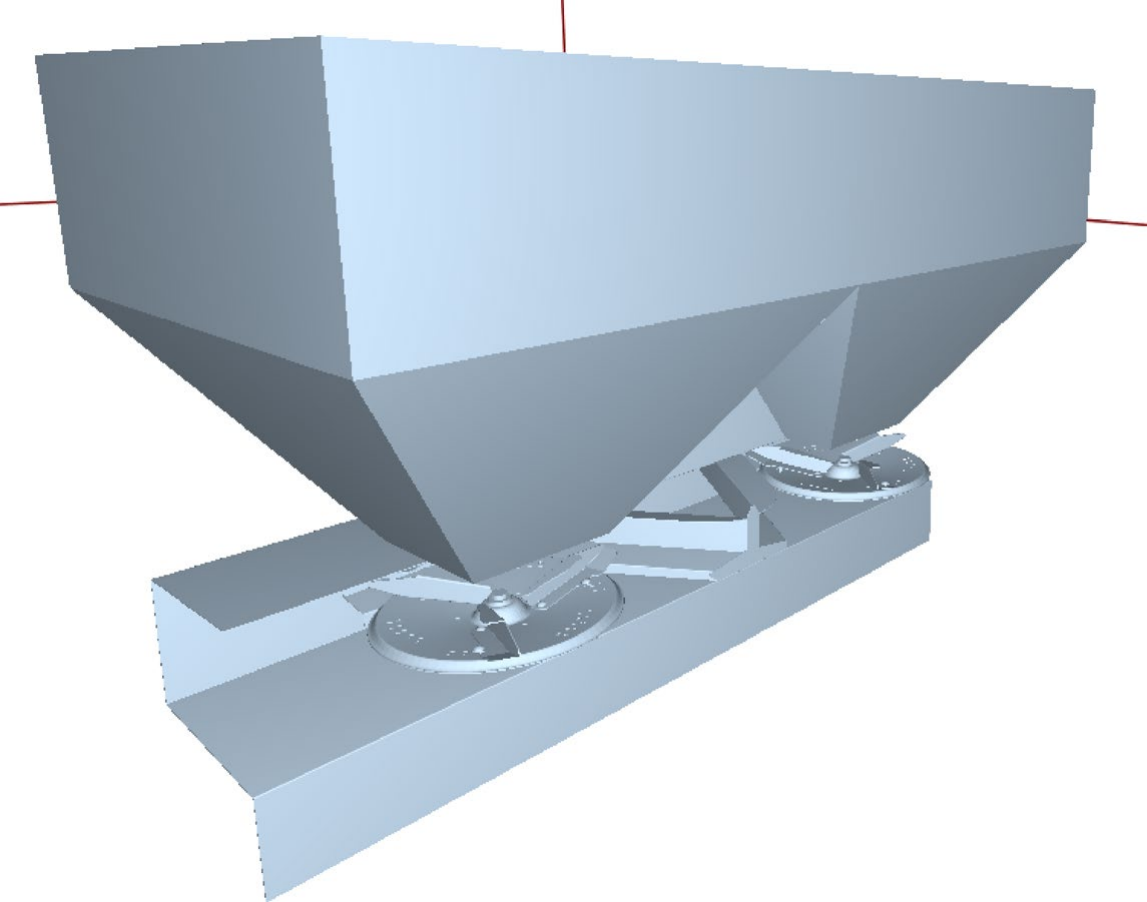
Transferred 3D model to Altair EDEM software.

To perform DEM simulations, the materials of the fertiliser and machine parts were used in the software. In the first phase, the parametric three-dimensional model of the twin-disc fertiliser broadcaster was created (Fig. 4), and the prepared model was transferred in *.stl format (Fig. 5) to the solution environment of the Altair EDEM software^27^.

**Fig. 6:**
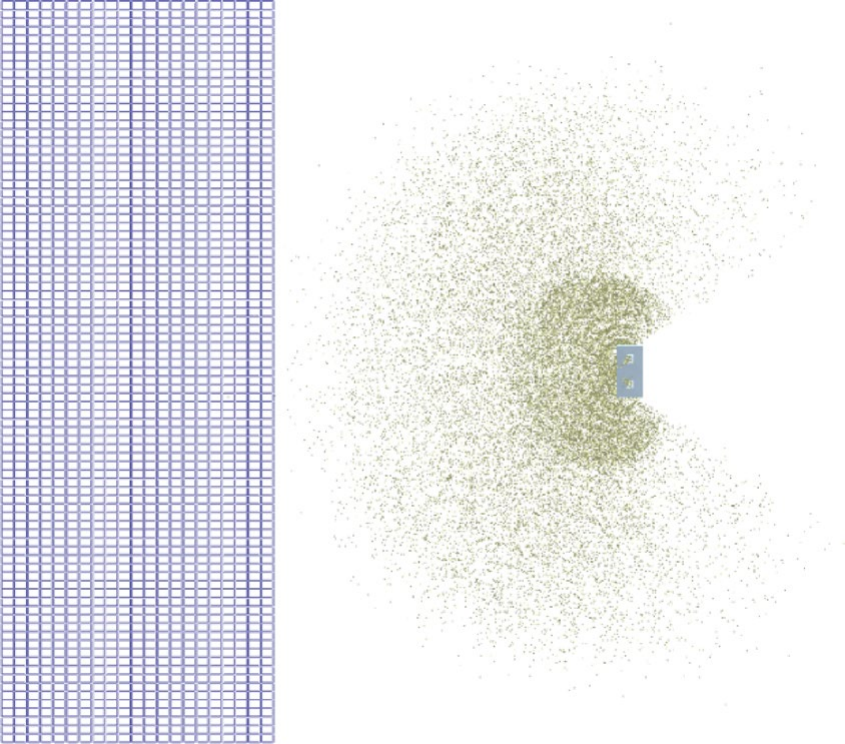
Simulation with a 21-row, 2-dimensional matrix for 11 s.

The fertiliser flow was set to a dynamically generated rate, and the simulation study was conducted at the same forward speed as used in the field tests of the centrifugal fertiliser broadcaster. The simulation period was set to 11 s. In the simulation analysis, the Hertz-Mindlin contact model and the Standard Rolling Friction model, both assumed to be non-slip, were used for particle-particle and particle-geometry interactions in the the Altair EDEM software^27^. The Schiller and Naumann drag model was selected for the particle-body force^28^. The properties of the medium through which the particles move after leaving the disc were assumed to be the specific gravity of air at 1.127 kg m^−3^ and the dynamic viscosity at 1.918 × 10^−5^ kg m^−1^ s^−1^ for the ambient temperature of 40 °C measured in the experiments. DEM simulation analyses were conducted in 21 rows in the form of a two-dimensional matrix on the field to ensure maximum repetitions in the forward direction during the 11-second simulation (Fig. 6).

To simulate the field tests dependent on granular material, the following material properties were specified: the dimensions of the granular fertilisers, the particle size distribution, the Poisson’s ratio, the shear modulus, the solid density, the coefficient of restitution, the coefficient of static friction, and the coefficient of rolling friction. Based on the results of the sieve analysis of the fertiliser used in the study (Table 1), the diameters of the fertiliser particles (0.5, 1.5, 2.675, and 3.675 mm) and their distribution in the Altair EDEM software were determined. In the Altair EDEM software, the fertiliser particles were normally distributed, with a standard deviation of 0.1. Poisson’s ratio, shear modulus, and solid density values for the fertiliser and machine material were taken from the literature (Table 2). Simulation studies were performed using the solid density value measured and declared by the domestic fertiliser producer, and the Poisson’s ratio^29^ and shear modulus^30^ values according to the type of fertiliser used.

**Table 2:** Poisson’s ratio, shear modulus, and solid density values for the fertiliser and machine material.

	Fertiliser	Steel
Poisson’s ratio	0.25^29^	0.3^21^
Shear modulus (MPa)	12.48^30^	7 × 10^4^^21^
Solid density (kgm^−3^)	1600 (measured)	7850^29^

Studies with different fertilisers and materials have shown that the coefficient of restitution, coefficient of static friction, and coefficient of rolling friction values, which determine the interaction of fertilisers with each other and with machine materials, vary widely^23,31–34^. Therefore, it was necessary to investigate the effects of these interactions on simulation experiments. In this context, screening experiments were conducted using the Plackett–Burman design to determine the effects of six different interaction values on fertiliser distribution evenness^31–34^. Coefficient of restitution (Fertiliser-Fertiliser), coefficient of restitution (Fertiliser-Steel), coefficient of rolling friction (Fertiliser-Fertiliser), coefficient of rolling friction (Fertiliser-Steel), coefficient of static friction (Fertiliser-Fertiliser) and coefficient of static friction (Fertiliser-Steel) were coded as “Rest_FF”, “StFri_FF”, “RollFri_FF”, “Rest_FS”, “StFri_FS”, “RollFri_FS” respectively. In these experiments, the independent variables were the six different interaction values, and the dependent variable was the deviation of the fertiliser distribution evenness in the simulation with a 16-m working width from the field experiments. The coded experimental design for Plackett–Burman is given in Table 3. The lower and upper limit values for the relevant variables taken from previous studies are given in Table 4^30–34^.

**Table 3:** The coded experimental design for Plackett–Burman.

Run order	Test factors					
	Rest_FF	StFri_FF	RollFri_FF	Rest_FS	StFri_FS	RollFri_FS
1	− 1	− 1	− 1	− 1	− 1	− 1
2	1	1	1	− 1	1	1
3	1	− 1	1	− 1	− 1	− 1
4	1	1	− 1	1	− 1	− 1
5	− 1	1	− 1	− 1	− 1	1
6	− 1	1	1	− 1	1	− 1
7	− 1	− 1	1	1	1	− 1
8	1	1	− 1	1	1	− 1
9	− 1	1	1	1	− 1	1
10	1	− 1	− 1	− 1	1	1
11	1	− 1	1	1	− 1	1
12	− 1	− 1	− 1	1	1	1

**Table 4:** The lower and upper limit values for the interactions.

Interactions	Lower limit	Upper limit	References
Rest_FF	0.1	0.4	^31,32^
StFri_FF	0.25	0.65	^32^
Roll_FF	0.1	0.35	^31,32^
Rest_FS	0.2	0.5	^35^
StFri_FS	0.2	0.55	^30,31^
Roll_FS	0.05	0.2	^30,34^

The results of the variance analysis of the Plackett–Burman design are given in Table 5. The independent variables accounted for 98.45% of the effect on the difference between field trials conducted at a disc peripheral speed of 16.26 m s^−1^ for the standard PTO speed and DEM simulations (y, %). However, in the field-simulation comparison, only StFri_FS was significant for material interaction values at α < 0.01. Based on these results, the Steepest Ascent test^34^ was used only for StFri_FS, and literature values were used for the other five variables. StFri_FS was subjected to simulation trials in the range 0–1 with a step value of 0.2. The comparison results obtained in simulation studies conducted at the disc peripheral speed determined in the study at standard PTO speed are shown in Table 6. To compare these results between simulation and field trials, the StFri_FS value of 0.6 was selected, which can be measured by friction tests and is within the wide range of values obtained for granular material. The data in Table 7 were used in the Altair EDEM software for simulations conducted with different disc peripheral speeds. The simulation time for each experiment was 11 s, with a target save interval of 0.01 s. During this period, between 55,000 and 62,000 particles were included in the model.

**Table 5:** Results of the variance analysis of the Plackett–Burman design.

Source	df	F (P*)
Rest_FF	1	7.77
		(0.039)
StFri_FF	1	2.18
		(0.2)
RollFri_FF	1	11.25
		(0.020)
Rest_FS	1	0.79
		(0.414)
StFri_FS	1	295.85
		(< 0,01)
RollFri_FS	1	0.28
		(0.618)
Error	5	
Total	11	

*P value < 0.01 indicates highly significant difference.

**Table 6:** The comparison results for steepest ascent test.

Run order	Rest_FF	StFri_FF	RollFri_FF	Rest_FF	StFri_FF	RollFri_FF	y (%)
1	0.32	0.38	0.15	0.42	0	0.1	− 24
2	0.32	0.38	0.15	0.42	0.2	0.1	− 23
3	0.32	0.38	0.15	0.42	0.4	0.1	− 10
4	0.32	0.38	0.15	0.42	0.6	0.1	0
5	0.32	0.38	0.15	0.42	0.8	0.1	3
6	0.32	0.38	0.15	0.42	1	0.1	4

**Table 7:** The coefficient values used in the DEM simulations.

Interactions	Fertiliser-fertiliser	Fertiliser-steel
Coefficient of restitution	0.32	0.42
Coefficient of static friction	0.38	0.60
Coefficient of rolling friction	0.15	0.10

## Results and discussion

**Fig. 7:**
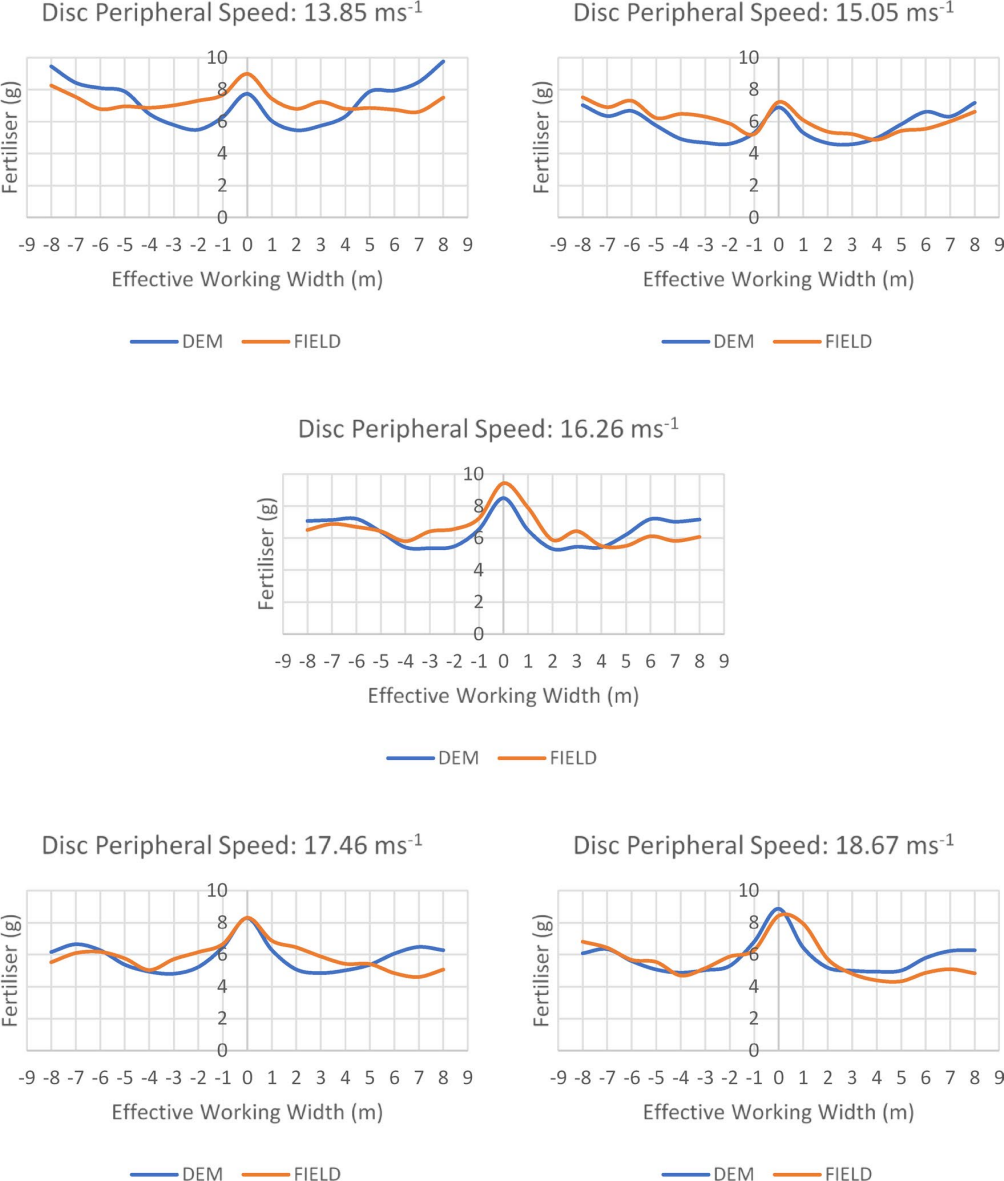
Comparison of DEM and field tests.

The diagrams in Fig. 7 show the results from a single pass of the fertiliser broadcaster. Comparing the values for the working width on the X-axis within the 16 m working area, between − 8 and + 8 m, illustrates the distribution of fertiliser in both the field tests and DEM simulations. Figure 7 presents a comparison of field tests and simulations conducted with a twin-disc fertiliser broadcaster equipped with three vanes, set at a fixed vane position angle of 43°, five disc circumferential speeds of 13.85, 15.05, 16.26, 17.46, and 18.67 m s^−1^, and a fixed fertiliser application rate. Table 8 provides a comparison between field and simulation experiments conducted with three vanes, showing differences in fertiliser quantities at a 16 m effective working width. The differences in fertiliser amounts at a 16 m effective working width range from 0 to 5.9%.

**Table 8:** Comparison between field and simulation experiments conducted with three Vanes at 16 m effective working width.

Disc peripheral speed (ms^−1^)	Difference of fertiliser amount between DEM simulation and field trials (%)
13.85	0.3
15.05	5.9
16.26	0.9
17.46	0.8
18.67	3.4

In this study, the maximum difference in fertiliser amounts at a 16 m effective working width is 5.9%. However, Yinyan et al.^21^ compared the distribution of potassium chloride fertiliser using a spreader operating on a similar principle to the machine used in this study in a single trial at a 300 g/s flow rate, 15° vane angle, 95 cm spreader disc height, and 600 rpm disc rotation speed in both simulation and field tests, determining a relative error of 10.66%. Similar to Yinyan et al.^21^ and Bivainis et al.^23^ found the greatest difference in fertiliser amount between field and simulation tests to be 9.87% under different application norms when spreading organic pellet fertiliser with a spreader operating on a principle similar to the machine used in this study. Examination of Fig. 7; Table 8 indicates that it is possible to use simulations instead of field trials when the properties of the fertiliser and twin-disc fertiliser broadcaster are accurately represented in the Altair EDEM software.

Van Liederke et al.^13^ compared a compound fertiliser using a single-disc fertiliser spreader with a working width of 3.5 m and reported that the distribution density in transverse distribution increased with increasing disc rotation speed as the distance from the feed line increased. In contrast, Fig. 7 shows that an increase in disc peripheral speed resulted in a decrease in fertiliser distribution density.

## Conclusions

For distribution tests conducted in field conditions with disc fertiliser broadcasters to comply with the ASAE 341.4 standard, it is necessary to operate under suitable weather conditions, which restricts the available time for testing. Additionally, these tests are demanding in terms of labour, cost, and time. The aim of this study is to demonstrate that results closely reflecting real conditions can be obtained if the characteristics of the machine and the fertiliser used in distribution tests with disc fertiliser broadcasters are entered into the Altair EDEM software, and the simulation parameters are adjusted to match field working conditions. The results of this study indicate that when researchers wish to determine the effects of structural or operational features of twin-disc fertiliser broadcasters on distribution, or to assess performance with different fertilisers, it is possible to establish and validate DEM simulation conditions in the field, rather than conducting all trials under field conditions. The key to successful simulation is to input realistic values for forward speed, fertiliser flow rate, contact models, rolling friction and drag, specific gravity, dynamic viscosity, and air temperature into the Altair EDEM software. This approach accelerates development cycles, reduces costs, and improves fertiliser distribution, thereby enhancing nutrient use and reducing environmental impact. Beyond twin-disc machines, these methods can be applied to other agricultural machinery and integrated into precision farming technologies, advancing sustainable, data-driven, and cost-effective solutions for modern agriculture.

## Data Availability

The data that support the findings of this study are available from the corresponding author, Fırat Kömekçi, upon reasonable request.
